# The looks of an odour - Visualising neural odour response patterns in real time

**DOI:** 10.1186/1471-2105-14-S19-S6

**Published:** 2013-11-12

**Authors:** Martin Strauch, Clemens Müthing, Marc P Broeg, Paul Szyszka, Daniel Münch, Thomas Laudes, Oliver Deussen, Cosmas Giovanni Galizia, Dorit Merhof

**Affiliations:** 1 Interdisciplinary Center for Interactive Data Analysis, Modelling and Visual Exploration (INCIDE), University of Konstanz, 78457 Konstanz, Germany; 2 Neurobiology, University of Konstanz, 78457 Konstanz, Germany

## Abstract

**Background:**

Calcium imaging in insects reveals the neural response to odours, both at the receptor level on the antenna and in the antennal lobe, the first stage of olfactory information processing in the brain. Changes of intracellular calcium concentration in response to odour presentations can be observed by employing calcium-sensitive, fluorescent dyes. The response pattern across all recorded units is characteristic for the odour.

**Method:**

Previously, extraction of odour response patterns from calcium imaging movies was performed offline, after the experiment. We developed software to extract and to visualise odour response patterns in real time. An adaptive algorithm in combination with an implementation for the graphics processing unit enables fast processing of movie streams. Relying on correlations between pixels in the temporal domain, the calcium imaging movie can be segmented into regions that correspond to the neural units.

**Results:**

We applied our software to calcium imaging data recorded from the antennal lobe of the honeybee *Apis mellifera *and from the antenna of the fruit fly *Drosophila melanogaster*. Evaluation on reference data showed results comparable to those obtained by previous offline methods while computation time was significantly lower. Demonstrating practical applicability, we employed the software in a real-time experiment, performing segmentation of glomeruli - the functional units of the honeybee antennal lobe - and visualisation of glomerular activity patterns.

**Conclusions:**

Real-time visualisation of odour response patterns expands the experimental repertoire targeted at understanding information processing in the honeybee antennal lobe. In interactive experiments, glomeruli can be selected for manipulation based on their present or past activity, or based on their anatomical position. Apart from supporting neurobiology, the software allows for utilising the insect antenna as a chemosensor, e.g. to detect or to classify odours.

## Introduction

### Motivation

Odours take many shapes, and equipped with an insect brain and a neuroimaging device one can reveal these shapes, turning chemicals into patterns and images.

In the conference version of this paper [1], we have introduced an imaging system that can read out and process brain activity in real time, making the neural representations of odours accessible. The biological motivation is that access to ongoing brain activity is the basis for analysing storage and processing of information in the brain, observing, for example, the activity patterns in response to odour stimulation. In particular, transforming odours into patterns and images does not only benefit basic neuroscientific research: It also allows us to utilise a living organism with highly sensitive olfactory organs as a chemosensor, where the patterns and the distances between them contain information about odour identity and dissimilarity.

We consider two application scenarios for real-time visualisation of odours using insect brains. In insects, the first stage of odour perception is formed by the odour receptor neurons on the antenna. Here, we utilise calcium imaging to record from the antenna of the fruit fly *Drosophila melanogaster*. While such data provides only little information about signal processing in the brain, receptor neurons on the antenna are easy to access experimentally and they are excellent chemosensing devices. As such they are a promising alternative to artificial chemosensors, also referred to as electronic noses (see e.g. [2-5]), that find application in environmental monitoring, chemical industry or security.

The second stage of odour processing in the insect brain is the antennal lobe (AL), a dedicated olfactory center where odours are represented by activity patterns of neural units, the so-called glomeruli [6]. A network of interneurons connects the glomeruli, and unravelling the function of this network in processing odour information is the topic of ongoing research. The honeybee AL is an established model for studying odour learning and memory [7], and neuropharmacological tools [8,9] have been developed to manipulate the network of interneurons. Here, the real-time aspect of odour visualisation is especially relevant as decisions can be based on prior activity, targeting e.g. glomeruli that have previously been part of a response pattern.

**Figure 1:**
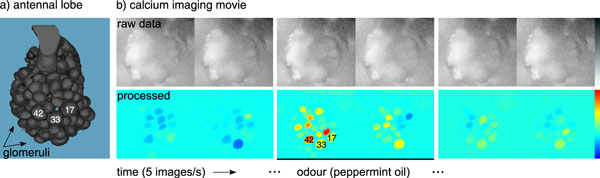
**Odour response patterns in the honeybee antennal lobe**. **a) **Anatomical model of the honeybee AL (modified from [55]). Landmark glomeruli (17, 33, 42) are labelled according to the numbering from [50]. **b) **Frontal view on the AL by calcium imaging. *Above: *Raw images (ratio: 340/380) from the movie. *Below: *Images processed with the real-time software. Glomeruli exhibit spontaneous background activity, or they respond to the odour during stimulus presentation (black bar).

From an image processing perspective, both application cases are similar. Activity patterns in the honeybee AL [10], and on the *Drosophila *antenna [11] are accessible through calcium imaging with fluorescent calcium reporters. Calcium imaging movies report fluorescence changes over time. Figure 1 gives an example for an imaging movie recorded from the honeybee AL, showing both the noisy raw images and processed, denoised versions of these images displayed in a false-colour scale.

The algorithm presented in this work computes a low-rank approximation based on few, selected (pixel) time series from the movie matrix. Exploiting the fact that there is noise and redundancy (time series from the same glomerulus are correlated) in the data, the movie matrix can be represented by another matrix of much lower rank. The rank-reduced version contains less noise, revealing the positions of the glomeruli and their signals [12]. As the glomerulus shapes become visible in Figure 1, such a representation may also be referred to as a segmentation, where the movie is segmented into regions with correlated activity over time, regions that correspond to the glomeruli.

Prior approaches to processing such imaging data involve manual feature selection [13,14], or, if automated, they can only be performed offline [12,15]. The real-time imaging system presented in this work allows for a wider range of applications, including closed-loop experiments, and it is what makes the readout from the antenna practical for chemosensing applications. Computation is performed in an adaptive manner, processing the movie stream incrementally as the images arrive from the camera. Fast processing is ensured by a GPGPU (General Purpose Computation on Graphics Processing Unit) implementation. The following sections provide further biological background and report on prior work. We then present algorithms and implementation details (Methods), followed by evaluation and demonstration of biological application cases (Results and discussion): Using the real-time imaging system, we visualise spontaneous activity and odour responses in the honeybee AL, and we provide a proof of concept for practical chemosensing with a biological sensor.

### Biological background

#### The olfactory system

The structure of olfactory systems is similar between insect species. As an example, we provide numbers for the honeybee *Apis mellifera*. On the antenna, approximately 60,000 odour receptor neurons interact physically with the odour molecules. These 60,000 neurons converge onto 160 glomeruli in the AL, where each glomerulus collects input from receptor neurons of one type. The glomeruli are bundles of synapses and appear as spherical structures with a diameter between 30 and 50 *µ*m. At the AL stage, each odour is represented by an activity pattern across the 160 glomeruli. Further upstream, this compact representation is widened again, as the projection neurons project from the glomeruli to approximately 160,000 Kenyon cells, a stage where odours are represented by sparser, high-dimensional patterns. [16,17] While implementation details differ between species, the combinatorial coding of odours by activity patterns across glomeruli, in the insect AL or in the vertebrate olfactory bulb, is a common feature of olfactory systems and can also be found in humans [18].

#### Odour responses in the AL

Calcium imaging using calcium-sensitive fluorescent dyes grants us access to the odour response patterns in the AL of the honeybee *Apis mellifera *[10]. These odour response patterns reflect the response properties of the odour receptor neurons, as well as additional processing that takes place in the AL. Interneurons connecting the glomeruli perform further computations such as contrast enhancement [10].

For an example of odour response patterns in the honeybee AL, see Figure 1. The activity pattern of glomeruli (between ca. 20 and 40 of the glomeruli are visible in an imaging movie) fluctuates at a low amplitude when no odour is present. After stimulation with an odour (indicated by the black bar), glomeruli exhibit individual responses to the odour. As a result, the activity pattern across glomeruli changes in way that is characteristic for the odour. The same odour elicits a similar pattern in different bees [6].

There is evidence that not only the identity of a particular odour is encoded by the corresponding glomerular response pattern, but that also chemical [19] and perceptual [20] (dis)similarity are reflected by the (dis)similarity of response patterns, suggesting that response pattern space is a rather faithful representation of chemical space.

#### Odour responses on the antenna

Glomerular response patterns, as measured in the honeybee recordings from this work, are the output signal of the AL, i.e. they are the result of integrating all receptor neurons of one type and of further processing that occurs in the AL network of interneurons. While odour coding is improved after this processing [21], the results of this paper suggest that chemical identity and (dis)similarity can already be inferred from receptor neuron signals recorded on the antenna, the earliest stage in the olfactory processing pipeline where response patterns are easily accessible without dissecting the brain.

In this work, antenna data was recorded in the fruit fly *Drosophila melanogaster*. The genetic tools that are available for *Drosophila *make it possible to express a calcium reporter directly in the receptor neurons on the antenna. Instead of expressing the reporter in cells of one type, as has been done before [11], the approach pursued here is to measure signals from a large set of different receptor cells that all express the general olfactory co-receptor Orco that these cells bear in addition to a specific odour receptor. This allows us to measure broad odour response patterns across many receptors. The segmentation approach presented in this paper is then used to to extract individual response units from the imaging movie based on their differential responses to a series of 32 different odours.

### Related work

Computational approaches to analysing imaging data can be classified as being either synthetic or analytic. In a synthetic approach, similar to common procedures for analysing fMRI data, Stetter et al. [22] have set up non-linear functions that they fitted to the individual (pixel) time series of the imaging movie. These functions can account e.g. for dye bleaching over time and for different neural signal components. Rather than performing bottom-up synthetic reconstruction of the imaging movie, analytic approaches decompose (bottom-down) the movie into factors. These are matrix factorisation or decomposition methods that exist in many different flavours, e.g. the well-known Principal Component Analysis (PCA). In particular Independent Component Analysis (ICA) has found widespread application on imaging data [15,23-26]. While ICA can be seen as a matrix decomposition method, the motivating paradigm for ICA is source separation. Under the assumption that there are underlying source signals that are statistically independent (and non-Gaussian), ICA algorithms (e.g. [27]) aim at recovering or separating these source signals on sample data where the sources appear in mixed form, e.g. neural signals mixed with measurement artifacts.

A recent convex analysis approach [12] (s.a. Methods), performs a factorisation of the movie matrix based on extremal column vectors from the boundary of the convex/conical hull of the data. Under the assumption that pure signal sources are present in the data, finding the extremal column vectors identifies these pure signal sources.

Traditionally, calcium imaging data from the insect AL has been processed by semi-automatic methods that perform e.g. image smoothing, but that still require human interaction to select regions of interest [13,14]. From the methods listed above, those that require human interaction appear less suited for real-time processing on a movie stream. So far, no real-time implementations of the computational approaches exist, the software implementations from [12,15] being only suited for offline data analysis.

## Methods

### Biological methods

#### Imaging the honeybee AL

For honeybees, it has been shown that projection neuron firing rate correlates with changes in intracellular calcium [28]. Staining with calcium-sensitive fluorescent dyes and excitation of the dyes with UV-light thus leads to a good proxy signal for brain activity [29].

**Figure 2:**
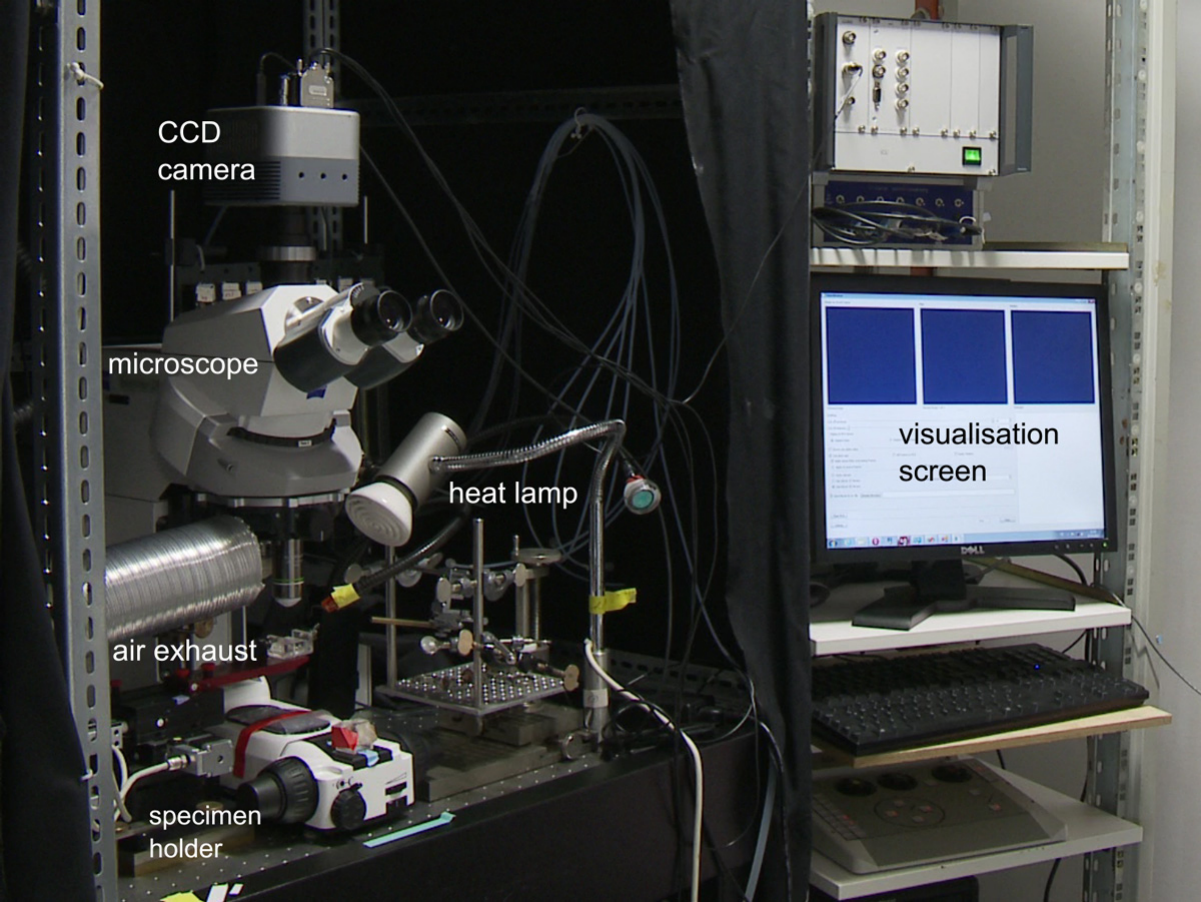
**Experimental setup for honeybee brain imaging**. Setup for the honeybee imaging experiments. After excitation with light at wavelengths 340 nm and 380 nm (light source not shown), fluorescence is recorded by a CCD camera mounted on top of a confocal microscope. Temperature is controlled by a heat lamp. The photograph shows the setup before the experiment, which is carried out in the dark. During the experiment, camera signals are processed in real time and results are displayed on the visualisation screen. Odour stimuli can be applied with a syringe (not shown), before being sucked out through the air exhaust.

Calcium imaging with forager honeyebees (*Apis mellifera*) was performed as described in [30]. Projection neurons in the l-APT and m-APT (lateral/medial antenno-protocerebral tract) were stained with Fura2-dextran (Invitrogen, Molecular Probes, Eugene, OR, USA), a calcium-sensitive, fluorescent dye. Activity of the projection neurons, that depart from the glomeruli in the AL, could be recorded using the experimental setup displayed in Figure 2. A fluorescence microscope (Axio Imager D.1, Zeiss, Göttingen, Germany) was equipped with a water immersion objective (20 ×, NA 0.95, Olympus, Tokyo, Japan). A light source (Polychrome V, TILL Photonics, Gräfelfing, Germany) provided excitation light at 340 and 380 nm, and fluorescence was recorded with a CCD camera (Andor Clara, Andor Technology PLC, Belfast, Northern Ireland). The input signal for data processing was computed as the ratio between consecutive images recorded at 340 and 380 nm, a standard procedure for Fura2-dextran [31].

#### Imaging the Drosophila antenna

##### Animals

Animals used for the experiments were female *Drosophila melanogaster *that were reared at 25 °C in a 12/12 light/dark cycle. Flies were of genotype w; P[Orco:Gal4]; P[UAS:GCaMP3]attP40, expressing the calcium reporter G-CaMP3 [32,33] in all Orco (olfactory co-receptor) bearing cells (UAS-GCaMP3 flies were provided by Loren L. Looger, Howard Hughes Medical Institute, Janelia Farm Research Campus, Ashburn, Virginia, USA).

##### Odorant preparation

Odorants were purchased from Sigma-Aldrich in the highest purity available. Pure substances were diluted in 5 mL mineral oil (Sigma-Aldrich, Steinheim, Germany) to a concentration of 10^-2 ^vol/vol. Odours were prepared in 20 mL headspace vials, covered with nitrogen and sealed with a Teflon septum (Axel Semrau, Germany). Odorants used were: 2-propylphenol (644-35-9), alpha-ionone (127-41-3), alpha-bisabolol (23089-26-1), trans-caryophyllene (87-44-5), (R)-carvone (6485-40-1), (S)-carvone (2244-16-8), beta-citronellol (106-22-9), 4-allyl-1,2-dimethoxybenzene (93-15-2), ethyl 3-hydroxyhexanoate (2305-25-1), ethyl (R)-3-hydroxybutanoate (24915-95-5), eugenol (97-53-0), E, E-farnesol (106-28-5), geraniol (106-24-1), heptyl acetate (112-06-1), hexyl acetate (142-92-7), hexyl butyrate (2639-63-6), isoamyl tiglate (41519-18-0), iso-eugenol (97-54-1), 4-isopropylbenzaldehyde (122-03-2), linalool (78-70-6), methyl 3-hydroxy hexanoate (21188-58-9), 4-methoxybenzaldehyde (123-11-5), methyl jasmonate (39924-52-2), (1R)-myrtenal (564-94-3), nonanal (124-19-6), nonanone (821-55-6), octyl acetate (112-14-1), phenylacetaldehyde (122-78-1), 4-hydroxy-3-methoxybenzaldehyde (121-33-5), gamma-propyl-gamma-butyrolactone (105-21-5), alpha-terpineol (10482-56-1) and alpha-thujone (546-80-5).

##### Stimulus application

A computer-controlled autosampler (PAL, CTC Switzerland) was used for automatic odour application. 2 mL of headspace was injected in two 1 mL portions at timepoints 6 s and 9 s with an injection speed of 1 mL/s into a continuous flow of purified air flowing at 60 mL/min. The stimulus was directed to the antenna of the animal via a Teflon tube (inner diameter 1 mm, length 38 cm).

The interstimulus interval was approximately 2 min. Solvent control and reference odorants (heptyl acetate and nonanone) were measured after every five stimuli (one block). The autosampler syringe was flushed with purified air for 30 s after each injection and washed with pentane (Merck, Darmstadt, Germany) automatically after each block of stimuli.

##### Calcium imaging

Calcium imaging was performed with a fluorescence microscope (BX51WI, Olympus, Tokyo, Japan) equipped with a 50x air lens (Olympus LM Plan FI 50x/0.5). A CCD camera (TILL Imago, TILL Photonics, Gräfelfing, Germany) was mounted on the microscope, recording with 4x4 pixel on-chip binning, resulting in 160x120 pixel sized images. For each stimulus, recordings of 20 s at a rate of 4 Hz were performed using TILL Vision (TILL Photonics, Gräfelfing, Germany).

A monochromator (Polychrome II, TILL Photonics, Gräfelfing, Germany) produced excitation light of 470 nm wavelength which was directed onto the antenna via a 500 nm low-pass filter and a 495 nm dichroic mirror. Emission light was filtered through a 505 nm high-pass emission filter.

Flies were mounted in custom-made holders, placed with their neck into a slit. The head was fixed to the holder with a drop of low-melting wax. A half electron microscopy grid was placed on top of the head, stabilising the antenna by touching the 2nd, but not the 3rd antennal segment.

### Matrix factorisation framework

**Figure 3:**
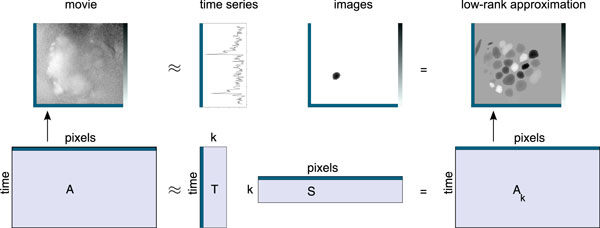
**Illustration of the matrix factorisation framework**. Matrix factorisation framework for imaging movies: The movie in matrix *A *is approximated by the product of the *k *time series in *T *and the *k *images in *S*, forming the rank-*k *matrix *A_k_*.

We first describe the general matrix factorisation framework for imaging movies. The framework is illustrated in Figure 3. An imaging movie can be cast into matrix form by flatting the two-dimensional images with *n *pixels into row vectors of length *n*. The movie matrix *A^m × n ^*has *m *time points and *n *pixels. The rows of the movie matrix, *A*_(*i*)_, contain images or time points. The columns, *A*^(*j*)^, contain pixels or time series.

We consider a factorisation of *A *into a matrix *T^m × k ^*of *k *time series and a matrix *S^k × n ^*of *k *images, where k≪n,m. This provides a low-rank approximation *A_k _*to the original matrix *A*:

(1) $$A^{m\times n}:A_{k}=T^{m\times k}S^{k\times n}=\overset{k}{\underset{r=1}{\sum }}T_{Ir}S_{rJ}$$

In imaging movies, all pixels that report the signal of the same glomerulus are correlated with each other (apart from the noise), which causes redundancy in *A*. It is thus possible to construct a good approximation with small *k*, such that ∥A-TS∥ is small.

The optimal rank-*k *approximation with respect to the aforementioned norm difference can be computed with Principal Component Analysis (PCA) [34]. However, the images in *S *computed by PCA are not sparse, with almost all pixels being different from zero [12]. The images in *S*, and the corresponding time series in *T*, can thus hardly be interpreted as the boundaries or the signal, respectively, of a particular neural unit. By definition, principal components need to be orthogonal to each other, which often prevents them from closely fitting the underlying source signals.

Ideally, as in the example from Figure 3, the images in *S *should be sparse, with only few pixels being different from zero. The *k *time series in *T *should be selected from *k *different glomeruli with the corresponding rows in the sparse *S *marking positions and boundaries of the glomeruli. We have shown in [12] that there is a method, the convex cone algorithm, that can achieve a factorisation with such favourable properties on imaging data.

### Convex cone algorithm

In this section, we review the convex cone algorithm from [12]. It is based on a non-negative mixture model for imaging data:

(2) $$A=TS^{0+}+N$$

The movie matrix *A *can be described by basis time series in *T *that are combined by coefficients in the non-negative matrix *S*^0+^. Residual noise is accounted for by *N*.

We assume that the columns *A*^(*j*) ^of the movie matrix contain either pure glomerulus signals or mixed signals, i.e. linear combinations (with non-negative coefficients) of the pure signals. At the fringes of a glomerulus, close to the neighbour glomeruli, such mixed signals can occur when a glomerulus signal is contaminated with additive light scatter from one or more neighbour glomeruli. Even if a glomerulus does not respond to an odour, light scatter can give the impression of a signal. In the middle of the glomeruli, that are rather large, circular objects, light scatter from the (distant) pixels of the neighbour glomeruli is less likely, and we assume that here the pixels contain pure signals.

For the matrix factorisation framework from Figure 3, we would like to select one pure signal from each glomerulus into *T*. Mixtures can then be modelled by *S*^0+^. While *S*^0+ ^can be computed easily given *A *and *T*, the challenging part is the selection of time series from the glomeruli into *T*.

Geometrically, the columns in *T *span a convex cone [35,36] that contains a part of the data points in *A*. Data points that lie within the cone can be reconstructed exactly by linear combination (with non-negative coefficients) of the columns in *T *. Data points that lie outside of the cone can be approximated by projecting them to the boundary of the cone, where the approximation error depends on the distance to the boundary.

From convex analysis we know that the set of extreme vectors of *A *is the minimal generator of the convex cone that contains the entire *A *[35,36]. With the extreme vectors we can span a volume that contains all data points of *A *and that thereby reduces the approximation error to zero. For imaging movies, the extreme columns vectors are also the columns with the pure signals from the middle of the glomeruli, whereas the mixed signal columns, that can be combined from the extreme, pure signal columns, lie within the cone.

Following this motivation, the convex cone algorithm [12] makes locally optimal choices for the next extreme column vector. With each new vector selected by the algorithm, the columns in *T *span a larger (≥) volume.

The convex cone algorithm starts with matrix *A*_{1} _:= *A*, selecting the column with index *p *that has the largest Euclidean norm: $\arg{\;\max}_{p}||A_{\left\{1\right\}}^{\left(p\right)}||$ This column becomes the first column of $T,T^{\left(1\right)}:=A_{\left\{1\right\}}^{\left(p\right)}$. Then, a matching $S_{\left(1\right)}:=A_{\left\{1\right\}}^{T}T^{\left(1\right)}$ is computed (for simplicity we omit the non-negativity constraint on *S*). The movie matrix is downdated as $A_{\left\{2\right\}}:=A_{\left\{1\right\}}-T^{\left(1\right)}S_{\left(1\right)}$. In the new matrix *A*_{2} _at iteration 2, the influence of the first column *T*^(1) ^is removed. We then select the column that is farthest away from the boundary of the cone, i.e. the column with the largest norm in *A*_{2}_. This is an estimate for the next extreme column vector, and we fill this column into *T*^(2)^.

We repeat the process until *c *columns are selected. In the following, we reserve *c *for the (user-specified) number of columns selected by the convex cone algorithm, and *k *for the number of principal components in the PCA step that is performed before the convex cone algorithm.

### Working on a movie stream

There are two motivations for performing PCA as a preprocessing prior to the convex cone algorithm. First, keeping only the top-*k *principal components reduces noise, which can make selection of extreme columns more robust. Second, we can utilise PCA to reduce computation time. For the real-time application, the movie matrix *A *grows by one row at each time point. The complexity of the convex cone algorithm is in the order O(mnc) if run once on the complete matrix *A*. For a growing movie matrix this would quickly accumulate a large overhead, the cost of performing the convex cone algorithm at each time point being O(1nc+2nc+…+mnc).

If we utilise PCA to keep, at all times, a compact summary matrix of constant size, we can remove the dependency on the growing time dimension. We propose to use an incremental PCA (IPCA) approach that computes the matrix *V_k _*of the top-*k *principal components at each time point, where *V_k _*is updated at low cost given the old version of *V_k _*and the current image received from the movie stream. The convex cone algorithm is then no longer performed directly on *A*, but on *V_k_*. As *V_k _*is the minimiser of $\left||A-V_{k}|\right|$, moderate values for *k *are sufficient in practice.

Several publications have treated IPCA algorithms [37-42]. Here, we rely on the CCIPCA algorithm by Weng et al. [39]. Several successful applications of CCIPCA can be demonstrated [43-45]. In these cases, CCIPCA was also used to incrementalise another algorithm by providing an updated version of matrix *V_k _*at each time point. CCIPCA costs a constant O(nk) operations per update, which amounts to O(mnk) for processing the entire movie once.

Here, we outline the basic principle behind CCIPCA. The first principal component is approximated as the mean of the images received so far. The second principal component is approximated as the mean of the images from which the projection onto the first PC has been subtracted, etc. This approach allows for incremental updates, and it completely avoids the time-consuming construction of a large *n *× *n *covariance matrix, which would be required by standard PCA approaches that compute the eigenvectors of the covariance matrix. In the following, we briefly outline the CCIPCA iteration. For further details on CCIPCA, see [39]. A convergence proof is given in [46].

We assume that the movie matrix grows by one image, *A*_(*i*)_, at time point *i*. The *r *=1*, ..., k *rows of the principal component matrix *V *= *V_k _*are initialised with *k *arbitrary, orthogonal vectors. Then, *V *is upated at each time point using the current image *A*_(*i*)_, where principal component *V*_(*r*) _at time point *i *is denoted $V_{\left(r\right)}^{\left\{i\right\}}$:

(3) $$V_{\left(r\right)}^{\left\{i\right\}}:=\frac{i-1}{i}V_{\left(r\right)}^{\left\{i-1\right\}}+\frac{1}{i}A_{\left(i\right)}A_{\left(i\right)}^{T}\frac{V_{\left(r\right)}^{\left\{i-1\right\}}}{∥V_{\left(r\right)}^{\left\{i-1\right\}}∥}$$

Then, image *A*_(*i*) _is downdated by subtracting the projection onto $V_{\left(r\right)}^{\left\{i\right\}}$:

(4) $$A_{\left(i\right)}:=A_{\left(i\right)}-A_{\left(i\right)}^{T}\frac{V_{\left(r\right)}^{\left\{i\right\}}}{∥V_{\left(r\right)}^{\left\{i\right\}}∥}\frac{V_{\left(r\right)}^{\left\{i\right\}}}{∥V_{\left(r\right)}^{\left\{i\right\}}∥}$$

After updating the *r*th principal component in this way, we can return to Equation (3) to update *V*_(*r*+1) _(Algorithm 1).

**Algorithm 1: ***V*^{*i*} ^= Update_IPCA (*V *^{*i*−1}^,*A*_(*i*)_, *k *, *i*)

**for all ***r *∈ [0, *k *− 1] **do**

$$V_{\left(r\right)}^{\left\{i\right\}}:=\frac{i-1}{i}V_{\left(r\right)}^{\left\{i-1\right\}}+\frac{1}{i}A_{\left(i\right)}A_{\left(i\right)}^{T}\frac{V_{\left(r\right)}^{\left\{i-1\right\}}}{∥V_{\left(r\right)}^{\left\{i-1\right\}}∥}$$

$$A_{\left(i\right)}:=A_{\left(i\right)}-A_{\left(i\right)}^{T}\frac{V_{\left(r\right)}^{\left\{i\right\}}}{∥V_{\left(r\right)}^{\left\{i\right\}}∥}\frac{V_{\left(r\right)}^{\left\{i\right\}}}{∥V_{\left(r\right)}^{\left\{i\right\}}∥}$$

end for

### Cone_updating: Visualisation in real time

Combining CCIPCA (Algorithm 1) and the convex cone algorithm leads to the algorithm at the core of the real-time imaging system: Cone_updating (Algorithm 2). Each image *A*(*i*) at time point *i *is first preprocessed by pixel-wise z-score normalisation: Subtract *µ*, the mean, and divide by *σ*, the standard deviation. Both, *µ *and *σ *of a pixel, can be updated as the movie stream proceeds.

After normalisation, the matrix *V *of the top-*k *principal components is updated with the current image: *V*^{*i*} ^:= Update_IPCA(*V*^{*i*−1}^, *A*(*i*), *k*, *i*). Finally, the Convex_cone_algorithm (*V*^{*i*}^, *c*) is applied to select *c *pixels (columns) from the current version of *V*.

As the movie matrix *A *grows, the incremental estimates for *µ*, *σ *and *V *improve. As a consequence, the *c *columns selected by the convex cone algorithm are better estimates of the extreme column vectors of *A*, the vectors that contain the pure glomerulus signals. In the matrix factorisation framework, these are are the columns for matrix *T*, and the corresponding *S *indicates glomerulus position (Figure 3).

Visualisations of brain activity, such as in Figure 1, can be achieved by low-rank approximation, using matrices T and S: *A_k _*= *TS*. At time point *i *we do not yet know the final *T *and *S*, and therefore we obtain the approximation ${\hat{A}}_{\left(i\right)}\;as{\hat{A}}_{\left(i\right)}:=A_{\left(i\right)}S^{\left\{i\right\}}S^{\left\{i\right\}}$, where $S^{\left\{i\right\}}$ is the current version of *S*.

For offline data visualisation, the colour scale can be adjusted to the maximum and minimum value of *A*. For real-time display, using one colour scale for the entire movie, maximum and minimum have to be updated incrementally. To avoid level changes, e.g. by long-term photobleaching of the calcium dye, data was high-pass filtered (0:025 Hz) before display in a false-colour scale (as in Figure 1).

**Algorithm 2 **: *S *= Cone_updating (*A*^(*m *× *n*)^, *c*, *k*)

  Initialise $V^{\left\{1\right\}}$

  **for all ***i *∈ [0, *m *- 1] **do**

    *A*_(*i*) _:= z_score_normalise (*A*_(*i*)_)

    **if ***i *> 1 **then**

      *V*^{*i*}^:= Update_IPCA(*V *^{*i*-1}^,*A*_(*i*)_, *k*, *i*)

      *S*^{*i*} ^:= Convex_cone_algorithm (*V*^{*i*}^, *c*)

${\hat{A}}_{\left(i\right)}:=A_{\left(i\right)}S^{\left\{i\right\}}S^{\left\{i\right\}}//$ low-rank approximation to image *A*_(*i*)_

    **end if**

  **end for**

### Implementations

We consider three implementations of the convex cone algorithm. Two implementations were written in Java, Java_offline, the reference implementation from [12], and Java_online. Java_offline performs exact offline PCA, followed by the convex cone algorithm, whereas Java_online (implementation of Algorithm 1) uses incremental PCA instead. Both Java implementations were performed in KNIME [47] (http://www.knime.org), a data pipelining environment.

Finally, we implemented the incremental online variant (Algorithm 1) using GPGPU: GPGPU_online. Z-score normalisation and the time-consuming PCA were implemented for the GPU with the NVIDIA CUDA [48] Basic Linear Algebra Subroutines (cuBLAS) (http://developer.nvidia.com/cublas) and the CUDA Linear Algebra library (CULA) (http://www.culatools.com/). The actual convex cone algorithm was run on the CPU.

TILL Photonics Live Acquisition (LA) Software 2.0 [49] was employed to control experimental hardware and excitation light intensity. GPGPU_online accessed the movie stream directly from the camera using a custom-built software interface kindly provided by TILL Photonics.

## Results and discussion

This sections starts with a technical evaluation of the proposed algorithm and a comparison of different implementations. We then demonstrate practical applicability in an experiment with honeybees and show how the techniques developed in this work can be utilised to turn the insect antenna into a living chemosensor. We conclude with a discussion regarding the impact that real-time processing of neural activity can have.

### Performance measures

#### Computing time

The motivations for adapting the matrix factorisation framework to the datastream domain were the ability to perform incremental updates upon arrival of new data, and of course the ability to process data with minimal time delay. For evaluation, we performed computation time measurements on a reference dataset. Measurements were carried out using an Intel Core i7 950 (3.07 GHz) CPU and a NVIDIA.

GeForce GTX 285 (648 MHz, 1024 MB) GPU. The Java_offline and Java_online implementations were run in KNIME (http://www.knime.org) workflows, and, for comparability with the C-implementation GPGPU_online, time measurements do only include the actual computation time and not the time for data transfer between nodes in the KNIME workflow.

The dataset consisted of 11 imaging movies of the honeybee AL (a part of the dataset was shown in [12]) with ≈ 170 × 130 pixels and ≈ 3500 time points each. The average length was about 15 minutes per movie. Table 1 reports overall computation time (in minutes) for the entire dataset and computation time per frame, averaged over all 11 movies. Both the incremental approximation to PCA and the GPGPU implementation contributed to the speedup. Java_online, that uses incremental PCA, achieved an approximately 1.5-fold speedup over Java_offline that is based on exact, conventional PCA. GPGPU_online achieved an additional 2-fold speedup over Java_online by using the GPU instead of the CPU. The parallelisation abilities of the GPU ensure scalability to future increases in data size, i.e. higher speedups are expected for data with higher resolution.

**Table 1:** Computation time measurements were performed on the 11 imaging movies of the reference dataset.

Implementation	(ms/frame)	overall time consumption (min)
Java_offline	134	68.15

Java_online	65	39.26

GPGPU_online	23	18.31


We report average computation time per frame and overall computation time for the entire dataset.

With the fastest implementation, GPGPU_online, a single image from the movie can be processed in 23 ms (Table 1), which is sufficient for calcium imaging in honeybees and *Drosophila *with typical recording frequencies below 20 Hz.

#### Approximation quality

**Figure 4:**
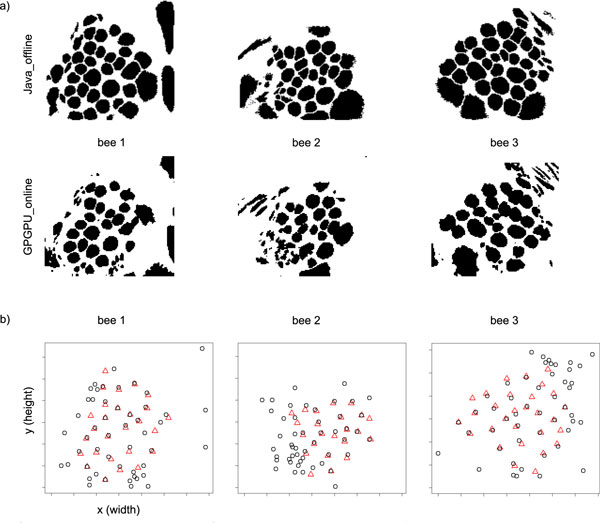
**Quality evaluation: Online implementation vs. offline reference implementation**. **a) **Comparing results of Java_offline and GPGPU_online using glomerulus maps for three bees (data from [12]). *Top: *Java_offline. *Bottom: *GPGPU_online. **b) **Positions of signals (columns) selected by the different implementations. Black circles: Positions of all *c *= 50 columns selected by GPGPU_online. Red triangles: Subset of signals (columns) selected by the reference implementation Java_offline. The subset contains only those signals that correspond to glomeruli. Glomeruli were identified based on their position in the maps, using the anatomical honeybee AL atlas [50].

The fastest implementation, GPGPU_online, achieves a significant speedup over the offline reference implementation. We next evaluated the quality of the results computed with GPGPU_online. There is an algorithmic approximation involved, and GPU computations are performed with float precision instead of double precision. In the online setting, incremental z-score normalisation was imperfect whenever there were mean shifts during the course of the experiment. How does this affect the quality of the results? For visualisation, we constructed maps of the glomeruli in the AL by overlaying all images from the rows of matrix *S*, the images that show the positions and boundaries of the glomeruli (see Figure 3). Such glomerular maps reveal the anatomy of the AL and can be matched between bees [12]. Using the reference results from [12], we compared glomerulus maps computed by Java_offline and GPGPU_online on the same movie. Parameters were *k *= 50 principal components, *c *= 50 (convex cone algorithm). Figure 4a shows glomerulus maps constructed by the two implementations. Clearly, both implementations reveal the same anatomy, but there is no perfect correspondence between the maps.

Apart from visual inspection of glomerulus maps, where cluster size has a strong impact, we also analysed how robust signal (column) selection was against incremental approximation. The convex cone algorithm selects extreme column vectors into matrix *T *that correspond to the pure signal sources. Matrix *S *is computed given *A *and *T *, and the rows of *S *reflect the distribution of similarity with the corresponding signal sources in *T *. This gives rise to the clusters of similar pixels in *S *and on the glomerulus map. Preprocessing, such as incremental z-score normalisation and incremental PCA, and a postprocessing step employed in [12] to remove the residual noise *N *(Equation 2) all have an impact on signal similarity and thus influence cluster size. This affects especially clusters that correspond to e.g. areas of similarly strong background staining, illumination artifacts etc. that do not have such clearly distinct signals as the glomeruli.

To evaluate signal selection independent of cluster size, we visualised the positions of the columns selected by the offline reference implementation Java_offline, along with the positions of the columns selected by GPGPU_online. For the reference implementation, we included only glomerular signals, i.e. those that could be identified by matching glomerulus maps to the anatomical honeybee AL atlas [50]. Figure 4b shows that the positions of signals selected by GPGPU_online (black circles) are in good correspondence with the glomerulus "targets" provided by Java_offline (red triangles). We conclude that selection of relevant signals, i.e. glomerular signals, is robust against incremental approximation in the online setting.

### Documentation of a real-time experiment in the honeybee AL

**Figure 5:**
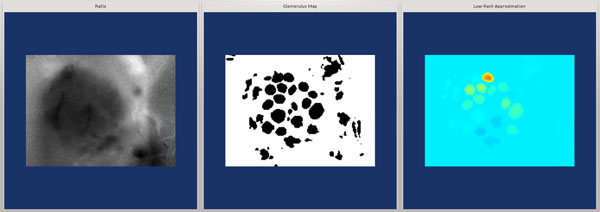
**Screenshot from the real-time software**. Screenshot. *Left*: Raw movie (fluorescence 340/380), *Middle: *Incrementally updated glomerulus map. *Right: *Low-rank approximation to the raw movie. We employ a min-max (blue-red) colour scale, where min and max are updated over the course of the experiment.

To demonstrate practical applicability, we performed a real-time experiment with honeybees. We used the experimental setup from Figure 2 and GPGPU_online, the software implementation that proved fastest in the evaluation. For a screenshot, see Figure 5. During the experiment, three windows were constantly updated: The raw fluorescence signal, shown as the ratio between consecutive measurements with 340 nm and 380 nm excitation light (see Methods), a map of the glomeruli in the AL, and the low-rank approximation to the current image. Movie documentations of the real-time experiment (Additional files 1 and 2) are available online.

**Figure 6:**
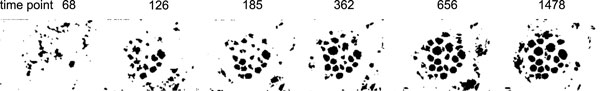
**Real-time experiment-Incremental map construction** . Development of a glomerulus map during a real-time experiment. The images show incremental updates of the map at different time points. Parameters: *k *= 50 (number of PCs), *c *= 50. As preprocessing, images were spatially filtered (Gaussian kernel, width = 9).

The glomerulus map is a segmentation of the image plane into regions with correlated activity over time. With the growing movie stream, more and more information about correlations between pixels becomes available. Figure 6 shows the gradual development of a glomerulus map during the course of the experiment. While at early time points many of the *c *basis signals were still influenced by the initialisation of the (not yet converged) incremental PCA (row of points in the left upper corner), they quickly moved to the positions of the glomeruli as more information arrived from the stream. Already after 400-600 time points, the map was almost complete. If needed, such an incremental computation of the glomerulus map could also be used to adapt to changes: For example, shifts between images caused by animal movement could be corrected for by giving higher weights to more recent time points.

**Figure 7:**
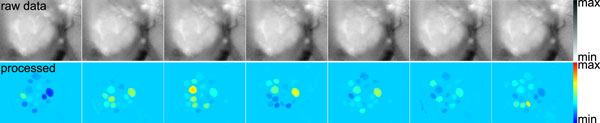
**Real-time experiment-Visualisation of spontaneous background activity** . Spontaneous background activity of glomeruli in the honeybee AL, visualised by low-rank approximation. *Top: *Fluorescence images recorded at 5 Hz. Each image is the ratio of consecutive images recorded with 340 nm and with 380 nm excitation light (see Methods). *Bottom: *The same images after processing with the real-time software.

Figure 1 visualised glomerular activity in response to odour stimulation. During the real-time experiment, we also observed spontaneous activity of glomeruli in the absence of odour stimulation, and also this spontaneous activity could be visualised by low-rank approximation: Figure 7. The ability to detect low-amplitude signals in spontaneous activity is relevant for the application scenarios discussed below (Impact of real-time processing).

### The insect antenna as a chemosensor

#### Functional segmentation of the antenna

While recording glomerular activity in the honeybee AL can help to answer neurobiological questions, the experimental procedure is technically demanding and time-consuming as it requires to dissect the brain and to fill in a calcium-dye into the projection neurons the day before the experiment. Working with the model organism *Drosophila melanogaster*, we could omit the manual staining step, genetically expressing the calcium-sensitive dye G-CaMP [32,51] in the receptor neurons that are accessible without dissecting the brain (see Methods).

This experimental design allows us to employ a *Drosophila *antenna as an easy-to-handle chemosensor. Similar to the situation in the honeybee AL, stimulating the antenna with a series of odour presentations elicits differential responses in the receptors that can then be distinguished based on their activity over time. We can thus utilise the real-time software to construct a map of the *Drosophila *antenna and to observe odour responses.

**Figure 8:**
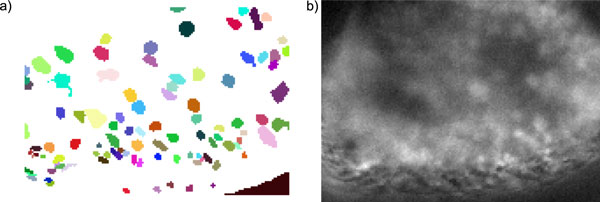
**Map of a Drosophila antenna**. Map of response units on the *Drosophila *antenna computed with GPGPU_online (*k *= 50, *c *= 100). **b) **Image from the antenna calcium imaging movie.

We employed a series of 32 different odour stimulations, two of which were control odours that were applied multiple times. This stimulation protocol elicited sufficiently diverse responses to allow for functional segmentation (with GPGPU_online). For Figure 8, we computed a map of the antenna consisting of *c *= 100 response units. The antenna map is shown in 8a, and Figure 8b contains a single image from the calcium imaging movie.

In *Drosophila*, olfactory receptor neurons are anisotropically spatially scattered over the surface of the antenna, so that odour response patterns form spatio-temporal maps. However, sources could be overlapping, creating a situation that is more complex than with the image of well segregated olfactory glomeruli in the antennal lobe.

#### Data analysis

**Figure 9:**
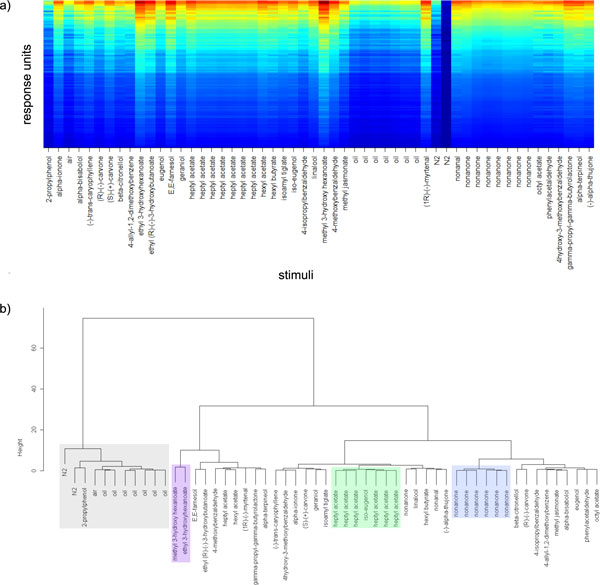
**Analysis of the Drosophila antenna recording**. **a) **Maximum response (after odour stimulation) for response units (y-axis) and a series of odour stimulations (x-axis). All odours were dissolved in (odourless) mineral oil, which was also given multiple times as a control. As a reference, the odours nonanone and heptyl acetate were applied multiple times. Response units are sorted by response to the first nonanone stimulation. Odours are sorted by name. The log colour scale ranges from blue (global min) to red (global max). **b) **Hierarchical clustering of the odour feature vectors from **a) **using Ward's method (stats package for R) based on Euclidean distances between the feature vectors. Marked clusters: Odourless substances (grey), hexanoates (purple), heptyl acetate (green), nonanone (blue).

We thus investigated whether the response units had indeed biologically or chemically meaningful signals. After processing the entire movie stream, we analysed the response unit time series in matrix *T *. For each odour stimulation, we computed a feature vector, where the feature was the maximum value of the response unit after presentation of the respective odour (and before the start of the next odour measurement): Figure 9a.

Clustering these feature vectors (Figure 9b) shows that feature vectors for repeated applications of the same odour, e.g. nonanone, cluster together. The odourless control measurements (mineral oil, air, N2) appear clearly separated from the odourous substances, and chemically similar odours end up in the same cluster (ethyl- and methyl-3-hydroxy-methanoate). This serves as a proof of concept, demonstrating that the real-time imaging system can, in principle, both recognise known odours and estimate the identity of unknown odours by their similarity to reference odours.

While distances between odour molecules are in part well reflected by response pattern distances, this is not always the case. For example, iso-eugenol does not fit into the heptyl acetate cluster, and 2-propylphenol lacks clear responses and therefore ends up in the (odourless) oil/air cluster. Further experiments are needed to evaluate whether representation of chemical identity can be optimised by recording more or different response units, e.g. in a different focal plane.

It also needs to be tested whether the observed odour responses are stereotypical across many individuals. As a first approach, we replicated the experiment from Figure 9, finding a high correlation (Pearson correlation 0.86, *p *=0.001, Mantel test for correlation of distance matrices) between the odour × odour Euclidean distance matrices (based on the feature vectors) from both experiments, indicating that the relative dissimilarity of odours could be conserved between individuals.

#### How can the system be applied?

Artificial chemosenors, so-called electronic noses [2-5] are important tools for environmental monitoring, healthcare or security applications. They do, however, not yet reach the efficiency and sensitivity of biological olfactory systems. The real-time software can extract features from calcium imaging recordings, directly accessing the *Drosophila *antenna as a biological chemosensor. Such feature vectors (Figure 9a) can be used to visualise molecular identity, or they can be subject to further processing, e.g. by classifiers, aiming to determine the identity of an unknown chemical substance. There are two points to making the biological chemosensor practical: 1) Working with non-invasive biological techniques that allow for easy handling of the flies, 2) Software that can process the continuous stream of odour plumes encountered in a real-world application.

### Impact of real-time processing

Going beyond the specific example of the chemosensor application, real-time processing has a wider range of applicability that involves any kind of interactive experimentation. This belongs to future work that is made possible with the real-time technology.

#### Motivating examples

It is increasingly clear that perception is influenced by both the stimulus and the prior state of the brain. For example, brain oscillations during the pre-stimulus interval influence how a human subject perceives an auditory stimulus in an experimental setup targeted at multimodal sensory integration [52]. Sensory experience without external stimulation, stemming only from the current state of the brain, is known from medical phenomena such as tinnitus [53].

For honeybees, there is first evidence in the direction that spontaneous background activity of the glomeruli in the AL carries information about odours that have been encountered recently: Glomerular activity patterns similar to a particular odour response pattern reverberate minutes after the actual response has been elicited by odour stimulation [54].

Considering the growing interest in ongoing brain activity, it is increasingly important to develop experimental strategies that allow stimulus presentations to be conditional on ongoing brain activity states. With the real-time methods presented in this work, glomeruli can be targeted because of their responses to odours or because they are part of reverberating patterns in spontaneous background activity.

Real-time processing is necessary to answer fundamental questions regarding the role of ongoing brain activity: Is it a side-effect that simply occurs as a consequence of neuron and network properties? Are patterns in spontaneous activity actually read out for further processing in the brain? In conditioning experiments [7], bees learn to associate an odour with a sugar reward. Can rewarding a pattern in spontaneous activity have the same effect as rewarding the actual odour?

#### Added value by real-time processing

From a biological perspective, the added value provided by the real-time software is that brain activity can be interpreted based on processed information. Only milliseconds after the activity occurs, we can regard not only raw pixel values, but anatomically distinct and identifiable units, the olfactory glomeruli in our case.

While analysis of neural data is often is performed pixel-wise (or voxel-wise), the brain encodes odours in patterns across glomeruli. Being able to work on a glomerulus level allows us to match the odour response patterns we observe with known response patterns from a database, which can reveal the chemical identity of the stimulus molecule. For spontaneous background activity, we can analyse the distribution of glomerular patterns that informs us about the state the antennal lobe network is in, i.e. the prior state that is relevant for how the stimulus will be perceived.

#### How fast is fast enough?

Closed loop experiments, where measured brain activity controls experimental settings, require that data processing is faster than recording speed. In calcium imaging experiments, images are often recorded at frequencies of 20 Hz or slower. Thus, any processing of 50 ms/frame or faster is appropriate. Recordings with voltage-sensitive dyes, for example, are generally useful at 50 Hz or faster: The fastest neuronal processes, the action potentials, have a duration of 1-3 ms, so recordings at 1000 Hz would be ideal. The current speed of 23 ms/frame (Table 1) is already getting close to the 50 Hz value, but it is still far from the ideal 1000 Hz.

For many experiments, fast processing is a requirement, e.g. if we wish to follow and react to fluctuations in spontaneous activity. For the chemosensor task, the advantage lies in the fact that we can directly query a biological chemosensor instead of waiting for results from post-hoc data analysis. Fast processing reduces the delay of the chemical analysis and allows for high-throughput assays.

## Conclusions

In the brain, odours are represented as activity patterns across many neurons. Calcium imaging is a technique that lends itself to extracting such activity patterns, as it allows to record many units simultaneously. So far, software for calcium imaging data has focussed on *offline *data processing [12-15]. The algorithms and software presented in this work process calcium imaging movies *online*, making the neural representations of odours accessible directly when they occur.

Algorithmically, we rely on a matrix factorisation that is updated with every new image that arrives from the movie stream. A low-rank approximation to the movie matrix serves as a compact representation of the calcium imaging movie, discarding noise and highlighting neural signals. This serves as the basis for further visualisations, such as functional maps of the glomeruli in the AL: Glomerulus borders are not defined by anatomy, but by function, i.e. activity (in response to odours) of pixels over time. This eliminates the need for registration of imaging data to anatomical stainings.

Such maps and the visualisation obtained by low-rank approximation reveal the "looks of an odour", the initial odour response pattern on the antenna, or, after data integration and processing has taken place, the glomerular response pattern in the AL.

Both odour representations have applications that profit from real-time processing. The role of the AL network in shaping the odour response patterns can now be investigated using closed-loop experiments, where prior system states influence current experimental parameters. Staining an array of receptor neurons with a single genetic construct, accompanied by online processing, provides easy access to odour response patterns, making real-time chemosensing with a biological sensor practical.

Visualising the neural representation of odours serves also to map perceptional spaces. Distances between odour response patterns are an estimate for perceptional (dis)similarity between odours [20] throughout different stages of odour information processing in the same individual, and also between individuals and even species, leading to species-specific odour perception spaces.

For such and further applications, the algorithmic and visualisation framework developed here enables fully automatic processing of odour response data without the need for human interaction to define e.g. regions of interest.

## Availability

Source code is available in Additional file 3.

## Competing interests

The authors declare that they have no competing interests.

## Authors' contributions

MS developed computational methods. MS wrote the manuscript with contributions from PS, DMü and MPB. CM (C++) and MS (Java) programmed software. CM, MPB and MS performed practical implementation and testing of the real-time imaging system. Biological experiments were performed by DMü and TL (*Drosophila*), and by PS (honeybee). CGG (biology) and DM (computer science) supervised the project. OD, CGG and DM revised the manuscript. All authors read and approved the final manuscript.

## Supplementary Material

Additional file 1 **Video documentation, part 1**. Experimental setup for honeybee brain imaging. Click here for file

Additional file 2 **Video documentation, part 2**. Screen capture from a honeybee brain imaging experiment. Click here for file

Additional file 3 **Source code**. Archive containing source code for the software presented in this work. Note that TILL Photonics LA 2.0 [49] is required for configuring experimental hardware. Click here for file

## Declarations

This publication was funded by the IEEE Symposium on Biological Data Visualization (BioVis) as a supplement of highlights.

The articles in this supplement have undergone the journal's standard peer review process for supplements. The Supplement Editors declare that they have no competing interests.

This article has been published as part of *BMC Bioinformatics *Volume 14 Supplement 19, 2013: Highlights from the 2nd IEEE Symposium on Biological Data Visualization. The full contents of the supplement are available online at http://www.biomedcentral.com/bmcbioinformatics/supplements/14/S19.
